# Factors influencing self-care among older patients with heart failure and spouses in China: a mixed methods study

**DOI:** 10.3389/fpubh.2026.1902052

**Published:** 2026-08-21

**Authors:** Xiaoyan Wu, Yiyu Zhuang, Manjun Wang, Jinhua Jin, Yuting Ke

**Affiliations:** Nursing Department, Sir Run Run Shaw Hospital, Zhejiang University School of Medicine, Hangzhou, China

**Keywords:** digital healthcare, dyadic, heart failure, mixed methods, self-care, social support, spouses

## Abstract

**Objective:**

This study aimed to investigate the factors influencing self-care among older patients with heart failure (HF) and their spouses from a dyadic perspective.

**Methods:**

An explanatory sequential mixed-methods design was employed. In the quantitative phase, 145 dyads of older HF patients and their spouses were recruited from a tertiary hospital in Zhejiang, China. Validated instruments were used to measure patient self-care, spouse contribution to self-care, disease-specific health literacy, mutuality, and social support. Multiple linear regression and the Actor-Partner Interdependence Model (APIM) were conducted separately for three self-care subscales (maintenance, management, confidence). In the qualitative phase, 10 dyads were purposively sampled for one-to-one semi-structured interviews, and data were analyzed via Colaizzi’s phenomenological method. Quantitative and qualitative findings were integrated adhering to the Theory of Dyadic Illness Management (TDIM).

**Results:**

Both patients and spouses scored below the 70-point adequacy threshold across all three self-care subscales. APIM revealed significant actor effects of health literacy and mutuality on self-care for both dyad members. Significant partner effects were observed in two dimensions: spouse mutuality was positively associated with patient self-care maintenance (*B* = 5.352, 95% CI: 0.631–9.828, *p* = 0.027), and spouse health literacy was positively associated with patient self-care confidence (*B* = 0.364, 95% CI: 0.193–0.533, *p* < 0.001). No significant partner effect was detected in the self-care management dimension, which showed suboptimal model fit and was thus interpreted as exploratory. No significant paths of traditional social support were retained in final models. Qualitative findings identified four thematic levels (individual, dyadic, family/social, cultural), supplementing contextual factors such as economic burden, reliance on digital healthcare services, and neglect of spousal caregivers. The integration provided a plausible contextual hypothesis for the null quantitative association between traditional social support and self-care.

**Conclusion:**

Health literacy and mutuality are core correlates of self-care among older Chinese HF patient-spouse dyads, with dimension-specific dyadic interaction patterns. Clinical interventions should develop tailored dyadic support strategies for distinct self-care dimensions, and pay attention to optimizing age-friendly digital healthcare services and improving caregiving culture. This study provides exploratory evidence for context-specific extension of the TDIM framework.

## 1 Introduction

Heart failure (HF) is a major public health challenge in China, characterized by high incidence, high prevalence, high mortality, high hospitalization rates, and a high economic burden. Driven by an aging population, the prevalence of HF in China is rising rapidly (1). There were approximately 14.3 million heart failure patients in 2023, representing a 208.4% increase from 1990 in China (2). While prevalence rates among young and middle-aged Chinese populations were comparable to global levels, rates among those aged 60 to 90 exceeded the global average for this age group (3). This trend is likely linked to specific national circumstances, such as the rapid pace of population aging and the fact that its long-term care management system remains underdeveloped. Despite advancements in heart failure treatment, outcomes remained suboptimal, characterized by a poor quality of life and higher mortality than that of cancer (4). HF patients lived with the disease for an extended period, experiencing physical discomfort, limitations in daily activities, and anxiety about the disease, which led to insomnia and depression (5). HF imposes a substantial economic burden on affected families and the broader healthcare system. These problems are prominent in older patients. Therefore, the population of older patients with HF and their disease management warrant attention.

In China, spouses serve as the primary informal caregivers for older HF patients. Older couples typically provide care for each other in later life. Spouses are not only accountable for patients’ care but also shoulder the family responsibilities previously shared by the patients. Spouses frequently confront exorbitant medical expenses, unpredictable fluctuations in the conditions, and disruptions to life balance, which can trigger negative emotions. Therefore, HF adversely affects the physical and mental well-being as well as the quality of life for the couples.

HF is a spontaneously progressive disease, and the primary therapeutic objectives are to alleviate symptoms, reduce readmission rates, and enhance the quality of life. Apart from medication and implantable device treatments, self-management, encompassing self-care maintenance, self-care monitoring, and self-care management, is vital for adults with HF (6, 7). Self-care behaviors included controlling water and sodium intake, participating in physical activity and exercise, restricting alcohol and tobacco consumption, monitoring body mass, and regulating psychological well-being (8). Self-care is the cornerstone of heart failure management (9). It was challenging for older HF patients to perform self-care behaviors, while caregivers played a crucial role in supporting patient self-care (10). Caregiver contributions to patients have been defined as the provision of time, effort, and support (11). Patients with caregivers’ support exhibit better adherence behaviors and are more likely to engage in self-care (12). However, self-care of older patients with HF and their spouses was notably inadequate (13).

In recent years, responses to chronic diseases have gradually shifted from the individual to the dyadic level, viewing illness as a dyadic event (14, 15). In intimate relationships, patients and their spouses are interdependent and mutually influential. When facing the changes and pressures brought by illness, both partners need to collaborate to cope. Higher patient-spouse mutuality contributed to better patient outcomes (16, 17). The Actor-Partner Interdependence Model (APIM) has been applied to explore self-care correlates. For instance, Jin et al. (18) used the APIM framework to examine health literacy and mobile health outcomes in Chinese chronic HF dyads, showing that improving the health literacy of patients and caregivers can encourage patients with CHF to use mobile health technologies, thereby enhancing their self-management ability. Kim et al. (19) investigated digital literacy and HF self-care among older patient-caregiver dyads and found predominantly actor effects rather than partner effects. However, existing studies remain limited: most rely on quantitative cross-sectional designs, lack integration of qualitative insights, and seldom address cultural factors specific to the Chinese context. To gain a more comprehensive understanding, this study employed an explanatory sequential mixed-methods approach. The aim of this study was to investigate individual, dyadic, social, and cultural determinants of self-care among older HF patient-spouse dyads, grounded in the Theory of Dyadic Illness Management (TDIM).

## 2 Materials and methods

### 2.1 Study design

This mixed methods study was reported in accordance with the Good Reporting of a Mixed Methods Study (GRAMMS) guidelines (20).

**Figure 1:**
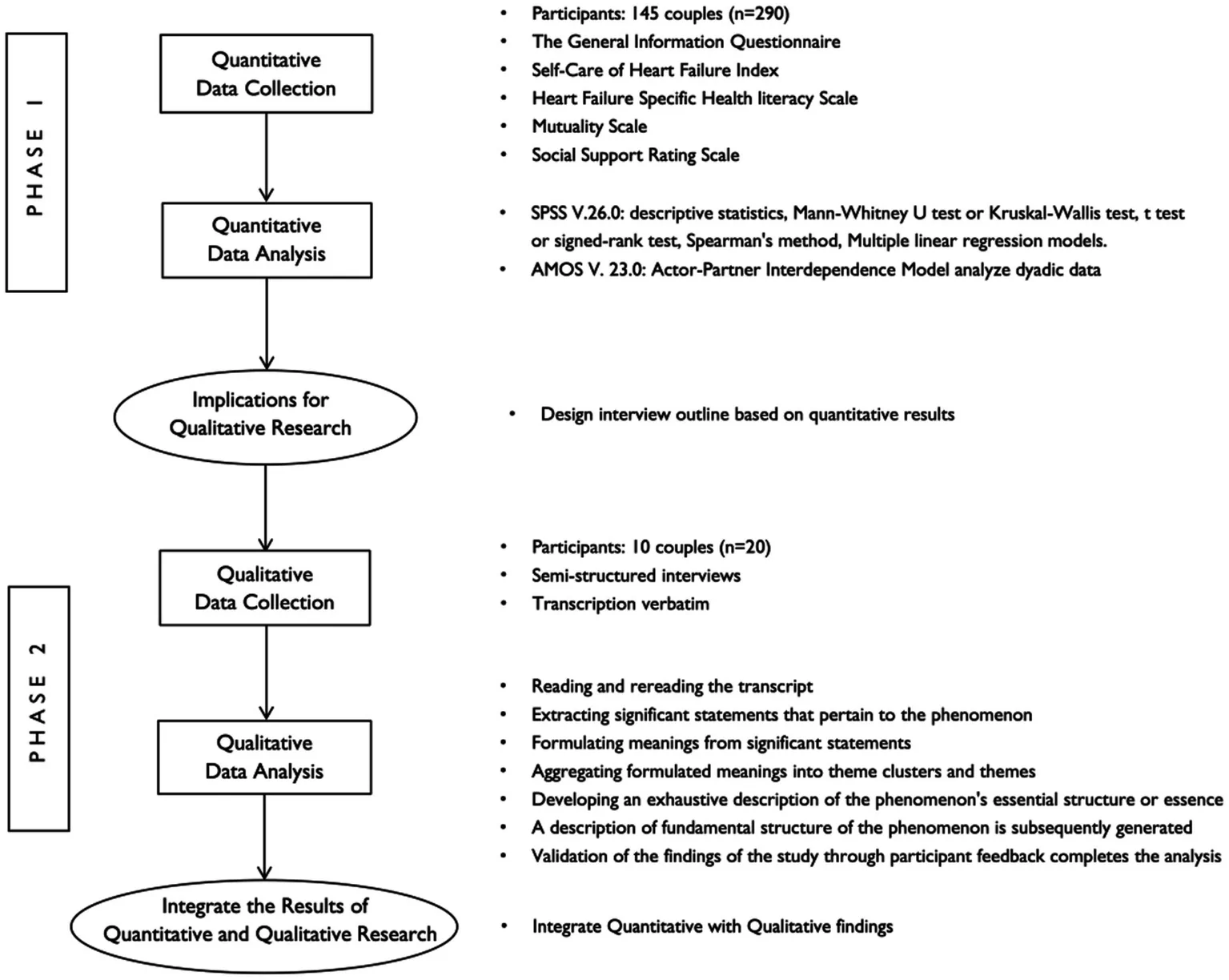
Methodological framework for the applied explanatory sequential mixed methods study.

The methodological framework in Figure 1 guided the research design. An explanatory sequential mixed-methods research design was adopted to enable the researchers to comprehensively explore the phenomena (21). An explanatory sequential mixed-methods approach typically begins with quantitative research to analyze variables and test hypotheses, and then, in response to statistical differences or clinically significant issues identified in the quantitative study, uses qualitative research to explore their meanings and seek explanations. This design is particularly suitable for investigating complex sociocultural factors in chronic disease management, as it combines the generalizability of sample surveys with the depth of phenomenological interviews. In this study, the quantitative phase was prioritized as the primary component. We first administered questionnaires to describe self-care status and identify statistically significant influencing factors. The qualitative phase was secondary and designed *a priori* to explain unexpected or ambiguous quantitative finding. The qualitative findings deepened and contextualized the quantitative results. The integration of the quantitative and qualitative data facilitated a more profound understanding of the self-care factors that affected older patients with HF and their spouses. The study was conducted between February and July 2024.

### 2.2 Theoretical perspective

TDIM was introduced by Lyons and Lee (22). The basic principle of the theory is that illness management is a dyadic phenomenon; it emphasizes the dyad as an interdependent team. The theory posits that dyadic illness management is influenced by contextual factors, including individual, dyad, family/social, and cultural aspects.

### 2.3 Phase 1: Quantitative study

#### 2.3.1 Participants and setting

From February to May in 2024, data were collected at a large tertiary general hospital in Zhejiang Province, China. The study site was a center of excellence for cardiology, providing medical service for people requiring cardiac therapy and care.

Inclusion criteria as follows. Patients: (1) Age ≥60 (according to the World Health Organization, older adults are typically defined as individuals aged 60 years and above); (2) married; (3) diagnosed with heart failure, New York Heart Association classification (NYHA) II-IV; (4) communicated normally; (5) voluntarily signed the informed consent. Spouse: (1) married; (2) caregiver of the patient; (3) communicated normally; (4) voluntarily signed the informed consent. Exclusion Criteria as follows. Patients: (1) diagnosed with dementia or cognitive impairment; (2) diagnosed with acute heart failure; (3) diagnosed with serious diseases such as cerebrovascular and tumor. Spouse: (1) diagnosed with dementia or cognitive impairment; (2) diagnosed with serious diseases such as cerebrovascular and tumor.

Sample size justification: The sample size for multiple linear regression was estimated based on a medium effect size (*f^2^* = 0.15), *α* = 0.05, and a statistical power of 0.80. With a maximum of 12 predictor parameters included in the primary covariate model, the required sample size was approximately 127 cases; this study enrolled 145 patient-spouse dyads, thereby meeting the basic requirements for testing the overall model. As the statistical power for APIM partner effects cannot be determined simply by the sample size-to-parameter ratio, a Monte Carlo sensitivity analysis (5,000 iterations) was additionally conducted based on the observed correlation structure of predictors, the residual variance of the final model, and a two-sided *α* of 0.05. The results indicated that, with a sample of 145 dyads achieving 80% power, the minimum absolute values of standardized partner effects detectable in the self-care maintenance, self-care management, and self-care confidence models were 0.30, 0.32, and 0.19, respectively; smaller partner effects falling below these thresholds might have been missed, and thus the APIM results are characterized as exploratory.

#### 2.3.2 Instruments

The demographic and clinical profile was collected using a standardized case report form. The patient’s form included age, gender, education, employment, medical insurance, marriage duration, financial pressures, times of hospitalization, self-care training experience and medical history, NYHA classification. NYHA classification was assessed by attending cardiologists at the time of study enrollment, based on the patient’s current cardiac function and symptom severity. The spouse’s form included age, gender, education, employment, marriage duration, financial pressures, self-care training experience and medical history.

The patient self-care was measured with Self-Care of Heart Failure Index, Version 6.2 (SCHFI 6.2) in three scales: self-care maintenance, self-care management, self-care confidence (23). Each subscale is scored independently in strict accordance with the developer’s scoring protocol. All items use a 4-point Likert response format. Raw item scores within each subscale are summed and converted to a standardized score ranging from 0 to 100. A standardized score below 70 is defined as inadequate self-care performance, and higher scores represent better self-care capacity in the corresponding dimension. Notably, the self-care management subscale is conceptually designed for respondents who have experienced HF symptoms during the specified recall period. All enrolled patients in this study were recruited from cardiology outpatient clinics and inpatient wards with confirmed NYHA functional class II–IV HF, and all reported typical HF symptoms (dyspnea and/or edema) within the 1-month recall window. Therefore, no “not applicable” responses were observed, and all 145 included patients were eligible for self-care management subscale scoring. Spouse contribution to patient self-care was measured with Caregiver Contributions to Self-Care of Heart Failure Index, Version 1 (CC-SCHFI 1), the content and scoring system are identical to those of the SCHFI 6.2 (11). In this study, the two scales showed high reliability. The Cronbach’s *α* for patient self-care maintenance, self-care management, and self-care confidence were 0.875, 0.907, and 0.935, while the McDonald’s *ω* were 0.903, 0.915, and 0.935. The Cronbach’s *α* for spouse contribution to self-care maintenance, self-care management, and self-care confidence were 0.876, 0.853, and 0.933, while the McDonald’s *ω* were 0.900, 0.867, and 0.930.

The patient health literacy was measured with Heart Failure Specific Health Literacy Scale (HFS-HLS) (24). It consisted of three subscales, namely functional health literacy, communicative health literacy, and critical health literacy. In this study, the Cronbach’s *α* of each subscale was 0.937, 0.846, and 0.916, while the McDonald’s *ω* were 0.945, 0.886, and 0.933. Each item was measured on a 4-point Likert scale, a higher score indicated higher level of health literacy. The spouse health literacy was measured with Health Literacy Scale for Chronic Heart Failure Caregivers (25). It contained three subscales of health knowledge and awareness, health behavior, and health skills, with Cronbach’s *α* of each subscale being 0.908, 0.928, and 0.937, while the McDonald’s *ω* were 0.899, 0.925, and 0.943 in this study. Each item was measured on a 5-point Likert scale, a score ≥105 was considered as indicating a high level of health literacy among caregivers. Notable differences exist between the two health literacy instruments in terms of dimensional structure and scoring. These two instruments were selected because they are the validated, disease-specific tools for their respective target populations in the Chinese context. However, they are not psychometrically equivalent, and direct cross-dyad comparison of raw scores should be approached with caution.

The relationship between the patient and spouse was measured with Mutuality Scale (MS), the scale comprised four dimensions: love and affection, reciprocity, sharing of pleasure, and values, each item was measured on a 5-point Likert scale. A score <2.5 was regarded as poor relationship (26). It demonstrated excellent internal consistency in this study: for patients, Cronbach’s *α* was 0.976 and McDonald’s *ω* was 0.977; for spouses, Cronbach’s *α* was 0.979 and McDonald’s *ω* was 0.980.

Social support was measured with Social Support Rating Scale (SSRS), the scale consisted of three dimensions: objective support, subjective support and utilization of support. A higher score indicated better social support received by the individual (27). In this study, SSRS suggested good reliability. For patients, Cronbach’s *α* was 0.749 and McDonald’s *ω* was 0.820; for spouses, Cronbach’s *α* was 0.778 and McDonald’s *ω* was 0.840.

#### 2.3.3 Data collection and analysis

Data collection: outpatient follow-up patients were selected at the end of their visit; hospitalized patients were selected 1 day before their discharge, older patients with HF and their spouses were surveyed separately. Of the 160 eligible patient-spouse dyads initially invited to participate, 8 dyads were excluded because one or both partners declined to participate. Among the remaining 152 dyads who returned their questionnaires, 7 dyads had incomplete responses with an item-level missing rate below 3%. Little’s Missing Completely at Random test confirmed that the missing data were completely random (*p* = 0.715), with no systematic missing pattern. Given that dyadic analyses require complete paired observations and that imputation may introduce bias in this dyadic context, we excluded these 7 dyads from the final analytical sample. The final sample consisted of 145 dyads with fully completed questionnaires (effective response rate: 145/160 = 90.6%).

Statistical analysis was performed using SPSS 26.0 and AMOS 23.0. The characteristics of the cohort and the items of the questionnaire were described using descriptive statistics, including mean and standard deviation (SD), or median and interquartile range (IQR) expressed as the 25th and 75th percentile for continuous variables, and comparisons between groups were made using the Mann–Whitney *U* test or the Kruskal-Wallis test, and paired comparisons were made using the paired-samples *t* test or the signed-rank test. Spearman’s method was used to analyze the correlation between quantitative variables. Internal consistency of the scales was assessed using Cronbach’s *α* and McDonald’s *ω*, calculated based on item-level data from 145 pairs of items according to the original scoring direction of the scale, with no post-hoc item deletion. The ω values were estimated using a one-factor homogeneous measurement model for each subscale.

Both multiple linear regression and APIM used the scores on the three subscales (self-care maintenance, self-care management, and self-care confidence) as dependent variables. For the multiple linear regression analysis, covariates were selected based on prior research, clinical significance, and the study’s theoretical framework. Adjustment variables were fixed prior to analysis and entered simultaneously using the Enter method. Variables representing employment and medical insurance type were excluded due to small subgroup sizes, thereby mitigating risks associated with unstable estimates for small categories and multicollinearity. Ultimately, the models for the three self-care subscales included 12 predictors: gender, age, education level, financial pressure, NYHA classification, time since heart failure diagnosis, times of hospitalization in the past year, other chronic diseases, self-care training experience, health literacy, mutuality, and social support. All models were assessed for linearity, normality of residuals, homoscedasticity, independence of residuals, multicollinearity, and influential outliers; heteroskedasticity-consistent covariance matrix estimator type 3 (HC3) robust standard errors, 95% confidence interval (95% CI), and exact *p*-values were reported consistently. Three dyadic models were constructed for the APIM, corresponding to self-care maintenance, management, and confidence. Core actor/partner paths comprised 12 paths linking health literacy, mutuality, and social support to the two outcomes of patients and spouses. APIM covariates were also pre-specified based on clinical and theoretical grounds, outcome equations adjusted for patient gender, age, education level, financial pressure, NYHA classification, time since heart failure diagnosis, times of hospitalization in the past year, other chronic diseases, self-care training experience. All covariate paths were retained without trimming based on *p*-values; only core actor/partner paths were removed stepwise based on the pre-specified criterion of *p* ≥ 0.05 and bootstrap 95% CI crossing zero. Given deviations from multivariate normality, the APIM utilized 5,000 dyadic bootstrap. Given that different measurement tools were adopted to assess health literacy in patients and spouses, three additional non-trimmed comparative models were constructed within each subscale APIM to examine whether the partner path of health literacy depended on the simultaneous inclusion of patient and spouse health literacy: a model including both patient and spouse health literacy, a model including only patient health literacy, and a model including only spouse health literacy. This analysis constituted a model specification sensitivity analysis. *p* < 0.05 was used to indicate that the difference was statistically significant. When *p* < 0.001, the result was reported as *p* < 0.001.

### 2.4 Phase 2: Qualitative study

#### 2.4.1 Participants

From June to July 2024, purposive sampling was employed to select interview participants with diverse characteristics for phase two of the study from among those who had already participated in phase one. Purposive sampling was guided by quantitative findings from phase one. Selection criteria included: (1) patients’ total score of self-care in low, medium, and high ranges of the distribution; (2) patients with different demographic and clinical characteristics (age, gender, health insurance type, financial strain, number of hospitalizations, NYHA classification, education level, employment status, and self-care training experience). The sample size was determined according to the principle of information saturation. No new themes emerged across the final two consecutive dyads, confirming that thematic saturation was achieved with the sample. Ultimately, 10 pairs of older patients with HF and their spouses were included in phase two, with patients labeled as P1-P10 and spouses labeled as S1-S10. The final 10 dyads covered all predefined demographic and clinical characteristics, achieving the intended maximum variation.

#### 2.4.2 The interview guide

The interview guide was developed based on the TDIM and an analysis of the data obtained in phase one of the study. The interview guide included six questions listed in Table 1.

**Table 1:** Guiding questions of the interview.

Number	Questions
1	When you learn that you (or your spouse) have chronic heart failure, how does it impact you?
2	What is your view on self-management? Is it solely the patient's concern, or a shared responsibility between the husband and wife?
3	Do you and your spouse have any disagreements during the process of managing illness?
4	Since you (or your spouse) have been ill, what factors have influenced the disease management?
5	Since the onset of the illness, apart from family, relatives, friends, and employer, what are the significant sources of support that have helped you fight it?
6	Since contracting the illness, which moments have made you feel challenged or fortunate? Could you offer some examples?

#### 2.4.3 Data collection and analysis

Patients and their spouses were interviewed separately in one-to-one sessions to enable each participant to express personal views freely, particularly on sensitive topics, and to capture convergent and divergent perceptions within the same dyad in a quiet rest area of the hospital. Six couples were interviewed following their outpatient follow-up consultations. For the other four couples, the patients were rehospitalized because their conditions worsened. Interviews were conducted 1 day before the patients’ discharge. Throughout the interview process, the researcher engaged with the interviewee using clear and comprehensible language, while maintaining a flexible and open demeanor. The researcher avoided any suggestion or leading of the interviewee, thus preserving objectivity and neutrality. Additionally, the researcher carefully documented paralinguistic cues throughout the interview, including tone of voice, facial expressions, emotional responses, and body language. The interview for each dyad lasted approximately 40 min.

All interviews were conducted by two researchers with master’s degrees in nursing. Both completed 8 h of specialized training in semi-structured interviewing skills. Neither interviewer was involved in the clinical care of the enrolled participants, which minimized positionality bias and created a neutral environment for participants to express their true views. After each interview, researchers promptly wrote interview logs and reflection logs, and returned the transcribed interview recordings to participants for verification. Sixteen of the 20 participants (80%) confirmed transcript accuracy. One spouse added a detail about online follow-up from community physicians, which was incorporated. The remaining four participants were unreachable. Member checking did not alter the core thematic structure, which had already reached saturation. Two independent researchers coded all verbatim transcripts line by line strictly following Colaizzi’s seven-step phenomenological method (28). The unit of coding was the individual participant. After coding all transcripts separately, the two researchers compared accounts within each dyad to identify convergent and divergent perspectives. Themes were subsequently classified as individual-level or dyadic-level based on whether they emerged from personal experiences or from the interplay between partners. For each dyad, interview responses were compared systematically. Convergent accounts were synthesized into shared themes, while divergent accounts were preserved as distinct findings and analyzed for their interpretive contribution to understanding dyadic dynamics. All coding discrepancies were resolved through in-depth discussion and final consensus with a third senior qualitative researcher with extensive phenomenological research experience.

### 2.5 Integration

The study employed an explanatory sequential design in mixed-methods research to gain a more in-depth understanding of the factors affecting self-care among older patients with HF and their spouses. Building upon the outcomes of the quantitative phase, the researchers conducted the qualitative phase to further investigate the relevant influencing factors. The findings were presented individually and then combined in a graph to corroborate and complement each other. Subsequently, the researchers integrated the qualitative and quantitative data to provide a comprehensive understanding of the factors influencing self-care in older patients with HF and their spouses.

### 2.6 Ethics

Ethical approval for the study was granted by the Medical Ethics Committee of the affiliated hospital of authors’ hospital (Approval Number: 20240432). All participants were provided with informed consent prior to their participation in the study. The data collected was anonymous and used solely for research purposes.

## 3 Results

### 3.1 Quantitative results

#### 3.1.1 Demographic results

A total of 160 dyads were invited, of which 145 dyads (90.6%) completed all questionnaires and were included in the final analysis. The respondents had a mean marriage duration of 41.78 ± 5.87 years (range: 26–57 years). The mean age of the patients was 65.68 ± 4.47 years (range: 60–78 years). The mean age of the spouses was 65.03 ± 5.08 years (range: 60–80 years). The patients’ mean diagnosis time of HF was 10.46 ± 5.65 months (range: 4–40 months). The demographic characteristics of the patients and spouses are presented in Table 2.

**Table 2:** Demographics of participants.

Predictor	Characteristic	Patients (*n* = 145)		Spouses (*n* = 145)	
		Frequency	Percentage (%)	Frequency	Percentage (%)
Gender	Male	81	55.86	64	44.14
	Female	64	44.14	81	55.86
Education level	Elementary	97	66.90	89	61.38
	Middle school	41	28.28	47	32.41
	College	7	4.83	9	6.21
Employment	Employed	5	3.45	6	4.14
	Retired	58	40.00	69	47.59
	Unemployed	4	2.76	4	2.76
	Farmers	78	53.79	66	45.52
Medical insurance	New rural cooperative medical insurance	81	55.86	-	-
	Basic medical insurance for employees	60	41.38	-	-
	Basic medical insurance for urban and residents	4	2.76	-	-
Financial pressure	Light	79	54.48	80	55.17
	Medium	44	30.34	42	28.97
	Heavy	22	15.17	23	15.86
Times of hospitalization	0	33	22.76	-	-
	1	72	49.66	-	-
	2	21	14.48	-	-
	≥ 3	19	13.10	-	-
NYHA classification	II	52	35.86	-	-
	III	59	40.69	-	-
	IV	34	23.45	-	-
Self-care training experience	No	135	93.10	135	93.10
	Yes	10	6.90	10	6.90
Other chronic diseases	No	29	20.00	77	53.10
	Yes	116	80.00	68	46.90

NYHA, New York Heart Association.

#### 3.1.2 HF self-care, health literacy, mutuality, and social support outcomes within dyads

All three subscales of self-care (maintenance, management, confidence) showed generally low performance among both older HF patients and their spouses, with all median subscale scores falling below 70 points. Patients scored significantly higher than their spouses across all three dimensions of self-care. The results are summarized in Table 3.

**Table 3:** Comparison of scores for patient self-care, spouse contribution to self-Care, health literacy, mutuality, and social support.

Item	Patient (*n* = 145)	Spouse (*n* = 145)	*Z*	*p*
Self-care maintenance	20.00 (13.33, 40.00)	16.66 (6.67, 36.66)	−6.667	<0.001
Self-care management	15.00 (0.00, 35.00)	10.00 (0.00, 25.00)	−3.904	<0.001
Self-care confidence	33.36 (16.68, 55.60)	27.80 (16.68, 55.60)	−2.599	0.009
Functional health literacy	7.00 (5.00, 10.00)	—	—	—
Communicative health literacy	8.00 (7.00, 11.00)	—	—	—
Critical health literacy	5.00 (4.00, 8.00)	—	—	—
Health knowledge and awareness	—	32.00 (28.00, 37.00)	—	—
Health behavior	—	26.00 (22.00, 33.00)	—	—
Health skills	—	31.00 (26.00, 36.00)	—	—
Total score of health literacy scale	20.00 (17.00, 29.00)	89.00 (74.00, 105.00)	—	—
love and affection	2.00 (2.00, 3.00)	2.00 (1.67, 3.00)	−2.164	0.030
Shared pleasurable activities	1.80 (1.00, 2.60)	1.80 (1.00, 2.80)	−0.573	0.567
Shared values	2.00 (1.50, 3.00)	2.00 (1.50, 3.00)	−0.113	0.910
Reciprocity	1.60 (1.00, 2.40)	1.60 (1.00, 2.40)	−0.630	0.529
Average total score of mutuality scale	1.87 (1.27, 2.67)	1.87 (1.27, 2.73)	−0.620	0.535
Objective support	9.00 (9.00, 10.00)	10.00 (9.00, 10.00)	−0.220	0.826
Subjective support	19.00 (17.00, 21.00)	19.00 (17.00, 21.00)	−1.305	0.192
Utilization of support	3.00 (3.00, 6.00)	4.00 (3.00, 6.00)	−0.206	0.836
Total score of social support scale	32.00 (29.00, 36.00)	32.00 (29.00, 37.00)	−0.921	0.357

Data are presented as M (P25, P75). Paired comparisons between patients and spouses were not performed for health literacy, because the dimensions and scoring ranges of the patient and spouse versions of the scale differed. The remaining paired comparisons adopted the Wilcoxon signed-rank test.

#### 3.1.3 Multiple linear regression analysis of factors influencing self-care among patients and spouses

In the enter regression models with prespecified covariates, patient self-care maintenance was negatively associated with age and positively associated with patient health literacy and patient mutuality. Patient self-care management was positively associated with NYHA classification, patient health literacy and patient mutuality. Patient self-care confidence was positively associated with patient health literacy and patient mutuality. Patient social support failed to reach statistical significance in all three fully adjusted models. Detailed analytical results for patients are shown in Tables 4–7. Spouse contribution to self-care maintenance was higher among female spouses, spouses with higher educational level, and dyads with higher patient NYHA classification. It was positively associated with spouse health literacy and spouse mutuality, yet negatively associated with spouse age. Spouse contribution to self-care management was positively associated with educational level, Self-care training experience patient NYHA classification, and spouse mutuality. Spouse contribution to self-care confidence was mainly positively associated with spouse health literacy and spouse mutuality. Spouse social support did not achieve statistical significance in any of the three fully adjusted models. Detailed analytical results for spouses are presented in Tables 8–11. All models met homoscedasticity and collinearity assumptions (VIF < 5). However, given the marginal significance of Ramsey’s RESET test for patient self-care management (*p* = 0.040) and spouse contribution to self-care confidence (*p* = 0.012), potential non-linearities should be acknowledged, and linear estimates cautiously interpreted as average linear trends, see Table 12.

**Table 4:** Omnibus test of regression models for the three patient subscales.

Outcome	Model *df*	Residual *df*	*F*	*p*	*R²*	Adjusted *R²*
Patient self-care maintenance	12	132	25.895	4.70×10⁻²⁹	0.702	0.675
Patient self-care management	12	132	22.258	3.51×10⁻²⁶	0.669	0.639
Patient self-care confidence	12	132	46.868	1.19×10⁻⁴¹	0.810	0.793

**Table 5:** Multiple linear regression analysis of patient self-care maintenance (HC3 robust standard errors).

Predictor	*B*	*SE*	95% CI	*t*	*p*	*VIF*
Constant	35.812	19.604	−2.967~74.590	1.827	0.070	—
Gender (Female)	1.131	2.170	−3.161~5.422	0.521	0.603	1.201
Age	−0.724	0.244	−1.206~−0.242	-2.969	0.004	1.331
Education level	2.118	2.763	−3.348~7.583	0.766	0.445	2.594
Financial pressure	−0.235	1.556	−3.313~2.842	-0.151	0.880	1.362
NYHA classification	3.040	1.726	−0.374~6.454	1.761	0.081	2.107
Time since heart failure diagnosis	−0.288	0.224	−0.732~0.155	-1.286	0.201	1.867
Times of hospitalization	−0.599	1.746	−4.052~2.853	-0.343	0.732	2.888
Other chronic diseases (Yes)	4.598	2.490	−0.327~9.523	1.847	0.067	1.226
Self-care training experience (Yes)	4.872	4.588	−4.204~13.949	1.062	0.290	1.212
Patient health literacy	0.705	0.237	0.235~1.174	2.971	0.004	3.559
Patient mutuality	11.618	1.917	7.826~15.411	6.060	1.33×10⁻⁸	2.185
Patient social support	−0.270	0.267	−0.798~0.258	-1.011	0.314	2.028

HC3, heteroskedasticity-consistent covariance matrix estimator type 3; *B* represents the unstandardized regression coefficient; *SE* represents the HC3 robust standard error; 95% CI represents the 95% confidence interval for *B*.

**Table 6:** Multiple linear regression analysis of patient self-care management (HC3 robust standard errors).

Predictor	*B*	*SE*	95% CI	*t*	*p*	*VIF*
Constant	−6.503	24.227	−54.426~41.419	−0.268	0.789	—
Gender (Female)	1.041	2.582	−4.066~6.149	0.403	0.687	1.201
Age	−0.442	0.294	−1.023~0.139	−1.504	0.135	1.331
Education level	2.335	3.166	−3.927~8.598	0.738	0.462	2.594
Financial pressure	1.748	1.739	−1.692~5.188	1.005	0.317	1.362
NYHA classification	8.453	2.111	4.277~12.629	4.004	1.03×10⁻⁴	2.107
Time since heart failure diagnosis	0.083	0.253	−0.417~0.582	0.327	0.744	1.867
Times of hospitalization	−0.987	2.285	−5.506~3.533	−0.432	0.667	2.888
Other chronic diseases (Yes)	5.271	2.682	−0.035~10.577	1.965	0.051	1.226
Self-care training experience (Yes)	5.281	3.981	−2.594~13.156	1.327	0.187	1.212
Patient health literacy	0.856	0.248	0.366~1.347	3.454	7.43×10⁻⁴	3.559
Patient mutuality	11.265	2.347	6.622~15.907	4.800	4.23×10⁻⁶	2.185
Patient social support	−0.366	0.367	−1.092~0.360	−0.997	0.321	2.028

HC3, heteroskedasticity-consistent covariance matrix estimator type 3; *B* represents the unstandardized regression coefficient; *SE* represents the HC3 robust standard error; 95% CI represents the 95% confidence interval for *B*.

**Table 7:** Multiple linear regression analysis of patient self-care confidence (HC3 robust standard errors).

Predictor	*B*	*SE*	95% CI	*t*	*p*	*VIF*
Constant	−5.263	23.155	−51.066~40.540	−0.227	0.821	—
Gender(Female)	0.094	1.978	−3.819~4.007	0.047	0.962	1.201
Age	−0.391	0.269	−0.923~0.142	−1.450	0.149	1.331
Education level	−2.239	2.626	−7.434~2.956	−0.852	0.396	2.594
Financial pressure	0.855	1.702	−2.513~4.222	0.502	0.617	1.362
NYHA classification	−0.622	1.726	−4.036~2.793	−0.360	0.719	2.107
Time since heart failure diagnosis	0.266	0.213	−0.155~0.687	1.248	0.214	1.867
Times of hospitalization	−1.234	1.885	−4.963~2.494	−0.655	0.514	2.888
Other chronic diseases (Yes)	0.168	2.301	−4.382~4.719	0.073	0.942	1.226
Self-care training experience(Yes)	−2.158	4.136	−10.338~6.023	−0.522	0.603	1.212
Patient health literacy	1.261	0.206	0.853~1.668	6.123	9.83×10⁻⁹	3.559
Patient mutuality	15.928	1.873	12.224~19.632	8.506	3.41×10⁻¹⁴	2.185
Patient social support	0.250	0.300	−0.344~0.844	0.833	0.406	2.028

HC3, heteroskedasticity-consistent covariance matrix estimator type 3; *B* represents the unstandardized regression coefficient; *SE* represents the HC3 robust standard error; 95% CI represents the 95% confidence interval for *B*.

**Table 8:** Omnibus test of regression models for the three spouse subscales.

Outcome	Model *df*	Residual *df*	*F*	*p*	*R²*	Adjusted *R²*
Spouse contribution to self-care maintenance	11	133	21.749	9.80×10⁻²⁵	0.643	0.613
Spouse contribution to self-care management	11	133	14.804	2.14×10⁻¹⁸	0.550	0.513
Spouse contribution to self-care confidence	11	133	33.033	8.50×10⁻³³	0.732	0.710

**Table 9:** Multiple linear regression analysis of spouse contribution to self-care maintenance (HC3 robust standard errors).

Predictor	*B*	*SE*	95% CI	*t*	*p*	*VIF*
Constant	9.784	17.456	−24.742~44.311	0.561	0.576	—
Gender (Female)	5.163	2.139	0.932~9.394	2.414	0.017	1.191
Age	−0.495	0.205	−0.902~−0.089	−2.411	0.017	1.325
Education level	6.617	2.754	1.171~12.064	2.403	0.018	2.577
Financial pressure	−1.132	1.336	−3.775~1.511	−0.847	0.399	1.225
Chronic diseases (Yes)	1.419	1.878	−2.296~5.134	0.756	0.451	1.086
Self-care training experience (Yes)	6.360	5.147	−3.821~16.541	1.236	0.219	1.324
Patient NYHA classification	3.800	1.706	0.425~7.175	2.227	0.028	1.942
Patient times of hospitalization	−2.302	1.600	−5.465~0.862	−1.439	0.153	2.103
Spouse health literacy	0.205	0.097	0.013~0.397	2.109	0.037	4.391
Spouse mutuality	8.570	1.654	5.298~11.842	5.180	8.01×10⁻⁷	2.132
Spouse social support	−0.212	0.274	−0.754~0.329	−0.775	0.439	2.196

HC3, heteroskedasticity-consistent covariance matrix estimator type 3; *B* represents the unstandardized regression coefficient; *SE* represents the HC3 robust standard error; 95% CI represents the 95% confidence interval for *B*.

**Table 10:** Multiple linear regression analysis of spouse contribution to self-care management (HC3 robust standard errors).

Predictor	*B*	*SE*	95% CI	*t*	*p*	*VIF*
Constant	2.371	18.193	−33.613~38.356	0.130	0.897	—
Gender (Female)	3.415	2.365	−1.262~8.092	1.444	0.151	1.191
Age	−0.435	0.226	−0.882~0.011	−1.929	0.056	1.325
Education level	5.879	2.819	0.303~11.454	2.086	0.039	2.577
Financial pressure	0.009	1.656	−3.266~3.285	0.006	0.995	1.225
Chronic diseases (Yes)	1.666	2.181	−2.648~5.979	0.764	0.446	1.086
Self-care training experience (Yes)	12.948	4.691	3.668~22.227	2.760	0.007	1.324
Patient NYHA classification	4.427	1.966	0.538~8.317	2.251	0.026	1.942
Patient times of hospitalization	−0.787	1.849	−4.444~2.870	-0.426	0.671	2.103
Spouse health literacy	0.080	0.117	−0.151~0.311	0.687	0.493	4.391
Spouse mutuality	7.761	1.821	4.160~11.363	4.263	3.80×10⁻⁵	2.132
Spouse social support	0.026	0.312	−0.591~0.643	0.083	0.934	2.196

HC3, heteroskedasticity-consistent covariance matrix estimator type 3; *B* represents the unstandardized regression coefficient; *SE* represents the HC3 robust standard error; 95% CI represents the 95% confidence interval for *B*.

**Table 11:** Multiple linear regression analysis of spouse contribution to self-care confidence (HC3 robust standard errors).

Predictor	*B*	*SE*	95%CI	*t*	*P*	*VIF*
Constant	−28.717	20.743	−69.747~12.312	−1.384	0.169	—
Gender (Female)	4.495	2.458	−0.367~9.357	1.829	0.070	1.191
Age	-0.190	0.228	−0.641~0.262	−0.832	0.407	1.325
Education level	3.228	2.546	−1.809~8.265	1.268	0.207	2.577
Financial pressure	−0.866	1.691	−4.211~2.480	−0.512	0.610	1.225
Chronic diseases (Yes)	−0.563	2.298	−5.109~3.982	−0.245	0.807	1.086
Self-care training experience (Yes)	−2.805	3.845	−10.410~4.799	−0.730	0.467	1.324
Patient NYHA classification	0.419	1.572	−2.689~3.528	0.267	0.790	1.942
Patient times of hospitalization	-0.791	1.811	−4.374~2.791	−0.437	0.663	2.103
Spouse health literacy	0.537	0.098	0.343~0.732	5.465	2.21×10⁻⁷	4.391
Spouse mutuality	10.692	2.020	6.696~14.688	5.293	4.84×10⁻⁷	2.132
Spouse social support	0.013	0.308	−0.597~0.623	0.042	0.967	2.196

HC3, heteroskedasticity-consistent covariance matrix estimator type 3; *B* represents the unstandardized regression coefficient; *SE* represents the HC3 robust standard error; 95% CI represents the 95% confidence interval for *B*.

**Table 12:** Assumptions and diagnostic results of six multiple linear regression models.

Model	Maximum *VIF*	*DW*	Residual S-W *P*	BP *P*	RESET *P*	Maximum Cook D	Maximum |Standardized Residual|	Treatment
Patient self-care maintenance	3.559	1.689	0.562	0.001	0.708	0.100	2.909	HC3 robust SE
Patient self-care management	3.559	1.622	0.059	0.016	0.040	0.065	3.107	HC3 robust SE; caution regarding functional form; several |standardized residual| >3, Cook D<1
Patient self-care confidence	3.559	1.389	0.768	0.049	0.608	0.103	3.343	HC3 robust SE; several |standardized residual| >3, Cook D<1
Spouse contribution to self-care maintenance	4.391	1.435	0.287	7.91×10⁻⁵	0.667	0.056	2.312	HC3 robust SE
Spouse contribution to self-care management	4.391	1.802	0.112	1.73×10⁻⁴	0.306	0.069	3.192	HC3 robust SE; several |standardized residual| >3, Cook D<1
Spouse contribution to self-care confidence	4.391	1.939	0.122	0.156	0.012	0.149	3.724	Homoscedasticity is generally acceptable; HC3 adopted uniformly; caution regarding functional form; several |standardized residual| >3, Cook D<1

VIF, Variance Inflation Factor; S-W test, Shapiro–Wilk test; BP test, Breusch–Pagan test; HC3, heteroskedasticity-consistent covariance matrix estimator type 3. *VIF* < 5 suggests no obvious multicollinearity; DW close to 2 indicates acceptable residual independence. A Breusch-Pagan test *P* < 0.05 indicates heteroscedasticity; a RESET test *p* < 0.05 implies potential misspecification of functional form. All Cook’s distance values were less than 1, with no influential observations detected. To maintain consistent inference, HC3 robust standard errors were reported for all six models.

#### 3.1.4 Actor-partner interdependence model analysis of self-care among patients and spouses

Mardia’s test for multivariate normality of the key continuous variables in the APIM is presented in Table 13. All 12 core paths are fully reported in Table 14, and transparent stepwise trimming was performed only on these core paths. When covariates were treated as fixed adjustment variables, the initial core path model was conditionally saturated (df = 0); thus, initial fit indices were not substantively interpreted. Model fit evaluation for the final models reflects the zero constraints imposed by the omitted core paths. The stepwise path trimming procedure for the three APIMs is shown in Table 15. The core path results of the final APIMs are detailed in Table 16. Fit indices of the initial and final APIMs are listed in Table 17. Monte Carlo sensitivity analysis of partner effects in the APIM based on the 145 dyads is presented in Table 18. Model specification sensitivity analysis with separate entry of health literacy variables is shown in Table 19. Results showed that in the self-care confidence model, when only spouse health literacy was entered into the model (*β* = 0.448, 95% CI: 0.319–0.568, *p* < 0.001), the direction and statistical significance remained stable, supporting the robustness of the reported partner path to specifications for health literacy entry. Evidence for health literacy partner effects was insufficient in the self-care maintenance and self-care management dimensions.

**Table 13:** Mardia’s test of multivariate normality for core continuous variables in APIM.

Model	Mardia skewness	*χ²*	df	*p*	Mardia kurtosis	*Z*	*P*
Self-care maintenance	8.086	195.416	120	1.65×10⁻⁵	82.805	1.335	0.182
Self-care management	9.434	227.979	120	1.04×10⁻⁸	84.228	2.012	0.044
Self-care confidence	8.177	197.609	120	1.05×10⁻⁵	87.399	3.522	4.29×10⁻⁴

APIM, Actor-Partner Interdependence Model. All three models departed from multivariate skewness, while the management and confidence models also deviated in multivariate kurtosis. Hence, 5000-sample dyadic bootstrap was used in the final APIM to derive path confidence intervals.

**Table 14:** All actor/partner paths in the three initial APIMs (2000 bootstrap samples for 95% CI).

Model	Outcome	Predictor	Effect	*B*	*β*	*SE*	Bootstrap 95% CI	*p*
Self-care maintenance	Patient self-care maintenance	Patient health literacy	Actor	0.565	0.249	0.271	0.105–1.136	0.039
		Patient mutuality		6.829	0.290	2.764	1.765–12.209	0.015
		Patient social support		−0.358	−0.095	0.326	−0.990–0.233	0.275
		Spouse health literacy	Partner	0.059	0.061	0.099	−0.139–0.253	0.557
		Spouse mutuality		4.716	0.208	2.669	−0.262–9.523	0.080
		Spouse social support		0.135	0.040	0.290	−0.405–0.711	0.642
	Spouse contribution to self-care maintenance	Patient health literacy	Partner	0.091	0.043	0.241	−0.351–0.569	0.708
		Patient mutuality		3.765	0.173	3.209	−2.350–9.576	0.243
		Patient social support		−0.528	−0.151	0.329	−1.116–0.156	0.111
		Spouse health literacy	Actor	0.203	0.228	0.119	−0.013–0.415	0.092
		Spouse mutuality		5.676	0.271	2.778	0.427–10.740	0.043
		Spouse social support		0.019	0.006	0.340	−0.616–0.652	0.956
Self-care management	Patient self-care management	Patient health literacy	Actor	1.057	0.398	0.330	0.480–1.712	0.002
		Patient mutuality		7.046	0.256	3.777	0.754–14.464	0.064
		Patient social support		−0.335	−0.076	0.458	−1.250–0.528	0.467
		Spouse health literacy	Partner	−0.228	−0.203	0.139	−0.492–0.028	0.104
		Spouse mutuality		5.502	0.208	3.312	−0.796–11.745	0.099
		Spouse social support		0.312	0.078	0.399	−0.458–1.032	0.436
	Spouse contribution to self-care management	Patient health literacy	Partner	0.238	0.113	0.275	−0.266–0.751	0.388
		Patient mutuality		4.337	0.198	3.482	−2.015–11.218	0.215
		Patient social support		−0.573	−0.163	0.400	−1.352–0.182	0.154
		Spouse health literacy	Actor	0.044	0.049	0.141	−0.213–0.318	0.757
		Spouse mutuality		4.316	0.204	3.197	−1.567–10.433	0.179
		Spouse social support		0.264	0.083	0.387	−0.436–0.989	0.496
Self-care confidence	Patient self-care confidence	Patient health literacy	Actor	0.792	0.280	0.243	0.304–1.241	0.001
		Patient mutuality		10.165	0.347	3.567	3.784–16.446	0.005
		Patient social support		−0.223	−0.047	0.399	−0.962–0.437	0.578
		Spouse health literacy	Partner	0.313	0.261	0.107	0.115–0.508	0.004
		Spouse mutuality		4.236	0.150	3.195	−1.314–10.196	0.187
		Spouse social support		0.380	0.089	0.327	−0.178–1.003	0.248
	Spouse contribution to self-care confidence	Patient health literacy	Partner	0.024	0.009	0.271	−0.482–0.538	0.928
		Patient mutuality		3.520	0.127	3.686	−3.556–10.244	0.341
		Patient social support		−0.314	−0.071	0.410	−1.068–0.455	0.445
		Spouse health literacy	Actor	0.542	0.477	0.120	0.311–0.773	1.40×10^−^⁵
		Spouse mutuality		8.008	0.300	3.332	1.567–14.015	0.018
		Spouse social support		0.126	0.031	0.395	−0.651–0.855	0.750

APIM, Actor-Partner Interdependence Model. The initial models are saturated models (df = 0). This table fully presents all 12 candidate actor/partner paths. Model fit is not evaluated based on fit indices of the initial models.

**Table 15:** Stepwise path trimming procedure for the three APIMs.

Model	Step	Outcome	Removed path (predictor)	*p* before removal	Rationale
Self-care maintenance	1	Spouse contribution to self-care maintenance	Spouse social support	0.956	Removal criterion: *p* ≥ 0.05 and bootstrap 95% CI spanning zero; covariates excluded from trimming.
	2		Patient health literacy	0.704	Removal criterion: *p* ≥ 0.05 and bootstrap 95% CI spanning zero; covariates excluded from trimming.
	3	Patient self-care maintenance	Spouse social support	0.642	Removal criterion: *p* ≥ 0.05 and bootstrap 95% CI spanning zero; covariates excluded from trimming.
	4		Spouse health literacy	0.505	Removal criterion: *p* ≥ 0.05 and bootstrap 95% CI spanning zero; covariates excluded from trimming.
	5		Patient social support	0.435	Removal criterion: *p* ≥ 0.05 and bootstrap 95% CI spanning zero; covariates excluded from trimming.
	6	Spouse contribution to self-care maintenance	Patient mutuality	0.211	Removal criterion: *p* ≥ 0.05 and bootstrap 95% CI spanning zero; covariates excluded from trimming.
	7		Patient social support	0.122	Removal criterion: *p* ≥ 0.05 and bootstrap 95% CI spanning zero; covariates excluded from trimming.
Self-care management	1	Spouse contribution to self-care management	Spouse health literacy	0.757	Removal criterion: *p* ≥ 0.05 and bootstrap 95% CI spanning zero; covariates excluded from trimming.
	2	Patient self-care management	Patient social support	0.467	Removal criterion: *p* ≥ 0.05 and bootstrap 95% CI spanning zero; covariates excluded from trimming.
	3		Spouse social support	0.707	Removal criterion: *p* ≥ 0.05 and bootstrap 95% CI spanning zero; covariates excluded from trimming.
	4	Spouse contribution to self-care management	Spouse social support	0.460	Removal criterion: *p* ≥ 0.05 and bootstrap 95% CI spanning zero; covariates excluded from trimming.
	5		Patient social support	0.239	Removal criterion: *p* ≥ 0.05 and bootstrap 95% CI spanning zero; covariates excluded from trimming.
	6		Patient mutuality	0.377	Removal criterion: *p* ≥ 0.05 and bootstrap 95% CI spanning zero; covariates excluded from trimming.
	7		Patient health literacy	0.168	Removal criterion: *p* ≥ 0.05 and bootstrap 95% CI spanning zero; covariates excluded from trimming.
	8	Patient self-care management	Spouse health literacy	0.092	Removal criterion: *p* ≥ 0.05 and bootstrap 95% CI spanning zero; covariates excluded from trimming.
	9		Spouse mutuality	0.151	Removal criterion: *p* ≥ 0.05 and bootstrap 95% CI spanning zero; covariates excluded from trimming.
Self-care confidence	1	Spouse contribution to self-care confidence	Patient health literacy	0.928	Removal criterion: *p* ≥ 0.05 and bootstrap 95% CI spanning zero; covariates excluded from trimming.
	2		Spouse social support	0.743	Removal criterion: *p* ≥ 0.05 and bootstrap 95% CI spanning zero; covariates excluded from trimming.
	3	Patient self-care confidence	Patient social support	0.578	Removal criterion: *p* ≥ 0.05 and bootstrap 95% CI spanning zero; covariates excluded from trimming.
	4	Spouse contribution to self-care confidence	Patient social support	0.473	Removal criterion: *p* ≥ 0.05 and bootstrap 95% CI spanning zero; covariates excluded from trimming.
	5		Patient mutuality	0.423	Removal criterion: *p* ≥ 0.05 and bootstrap 95% CI spanning zero; covariates excluded from trimming.
	6	Patient self-care confidence	Spouse social support	0.272	Removal criterion: *p* ≥ 0.05 and bootstrap 95% CI spanning zero; covariates excluded from trimming.
	7		Patient mutuality	0.204	Removal criterion: *p* ≥ 0.05 and bootstrap 95% CI spanning zero; covariates excluded from trimming.

APIM, Actor-Partner Interdependence Model.

**Table 16:** Paths of the three final APIMs (5000 bootstrap samples).

Model	Outcome	Predictor	Effect	*B*	*β*	*SE*	Bootstrap 95%CI	*p*
Self-care maintenance	Patient self-care maintenance	Patient health literacy	Actor	0.618	0.272	0.221	0.216–1.030	0.006
		Patient mutuality		6.167	0.262	2.552	1.409–11.179	0.017
		Spouse mutuality	Partner	5.352	0.236	2.394	0.631–9.828	0.027
	Spouse contribution to self-care maintenance	Spouse health literacy	Actor	0.175	0.197	0.088	0.009–0.345	0.049
		Spouse mutuality		8.454	0.403	1.629	5.423–11.599	7.65 × 10⁻⁷
Self-care management	Patient self-care management	Patient health literacy	Actor	0.820	0.309	0.233	0.388–1.272	5.87 × 10⁻⁴
		Patient mutuality		10.482	0.381	2.347	6.167–14.833	1.69 × 10⁻⁵
	Spouse contribution to self-care management	Spouse mutuality		8.615	0.407	1.649	5.500–11.689	6.44 × 10⁻⁷
Self-care confidence	Patient self-care confidence	Patient health literacy	Actor	0.798	0.282	0.235	0.346–1.224	9.09 × 10⁻⁴
		Patient mutuality		14.022	0.478	1.906	10.463–17.648	1.79 × 10⁻¹¹
		Spouse health literacy	Partner	0.364	0.303	0.092	0.193–0.533	1.22 × 10⁻⁴
	Spouse contribution to self-care confidence	Spouse health literacy	Actor	0.539	0.475	0.098	0.346–0.725	1.65 × 10⁻⁷
		Spouse mutuality		10.699	0.401	1.974	6.881–14.357	2.70 × 10⁻⁷

APIM, Actor-Partner Interdependence Model. The 95% CI denote 5000-sample dyadic bootstrap percentile confidence intervals for unstandardized coefficient *B*. All covariates were retained in corresponding outcome equations according to prespecified rules and were excluded from actor/partner path trimming. Only final core paths are presented in the table.

**Table 17:** Fit indices of the initial and final actor–partner interdependence models.

Outcome model	Stage	*χ²*	*df*	*P*	*RMSEA*	RMSEA 90%CI	*CFI*	*TLI*	*SRMR*
Self-care maintenance	Initial saturated model	0	0	—	—	—	—	—	Not evaluated
	Final parsimonious model	4.826	7	0.681	0.000	0.000-0.080	1.000	1.000	0.047
Self-care management	Initial saturated model	0	0	—	—	—	—	—	Not evaluated
	Final parsimonious model	20.459	9	0.015	0.094	0.039-0.149	0.873	0.831	0.049
Self-care confidence	Initial saturated model	0	0	—	—	—	—	—	Not evaluated
	Final parsimonious model	9.066	7	0.248	0.045	0.000-0.118	0.990	0.984	0.032

RMSEA, root mean square error of approximation; CFI, comparative fit index; TLI, Tucker-Lewis index; SRMR, standardized root mean square residual. When predefined covariates are treated as fixed adjustment variables, the initial core path model has df = 0, and the fit indices lack discriminative power. Conventional cutoff criteria for the final model are RMSEA <0.08, CFI/TLI >0.90, and SRMR <0.08. The RMSEA, CFI, and TLI of the self-care management model failed to meet the conventional thresholds, indicating inadequate fit of its parsimonious structure; the corresponding paths are interpreted for exploratory purposes only.

**Table 18:** Monte carlo sensitivity analysis for partner effects in APIM based on 145 dyads.

Model	Target partner path APIM	Number of dyads	Number of simulations	α	Minimum detectable |β| for 80% power
Self-care maintenance	Spouse mutuality → Patient self-care maintenance	145	5000	0.05	0.30
Self-care management	Spouse health literacy → Patient self-care management	145	5000	0.05	0.32
Self-care confidence	Spouse health literacy → Patient self-care confidence	145	5000	0.05	0.19

APIM, Actor-Partner Interdependence Model. The sensitivity analysis was conducted with 5000 simulations using the observed standardized predictor matrix and residual variances of the final equations. For the sample of 145 dyads under 80% statistical power, the minimum detectable absolute values of standardized partner effects were 0.30, 0.32, and 0.19 for the maintenance, management, and confidence models, respectively. Smaller partner effects may fail to be detected; therefore, the APIM analyses should be regarded as exploratory.

**Table 19:** Sensitivity analysis of model specification for Actor-Partner Interdependence Model with patient and spouse health literacy entered separately.

Subscale	Specification of health literacy entry into the model	Spouse health literacy → Patient outcome *β* (95% CI), *p*	Patient health literacy → Spouse outcome *β* (95% CI), *p*	*R²* of patient/Spouse equation
Self-care maintenance	Both entered simultaneously	0.031 (−0.156–0.208), 0.752	0.058(-0.153-0.274), 0.602	0.709/0.641
	Only patient health literacy	—	0.134(-0.039-0.311), 0.135	0.709/0.636
	Only spouse health literacy	0.161 (−0.002–0.313), 0.055	—	0.696/0.641
Self-care management	Both entered simultaneously	−0.168 (−0.386–0.035), 0.112	0.135(-0.104-0.379), 0.256	0.672/0.555
	Only patient health literacy	—	0.141 (−0.055–0.336), 0.144	0.666/0.555
	Only spouse health literacy	0.040 (−0.144–0.206), 0.678	—	0.639/0.550
Self-care confidence	Both entered simultaneously	0.303 (0.159–0.445), <0.001	0.022 (−0.164–0.205), 0.792	0.829/0.732
	Only patient health literacy	—	0.240 (0.072–0.404), 0.009	0.809/0.692
	Only spouse health literacy	0.448 (0.319–0.568), <0.001	—	0.813/0.732

Standardized *β*, 5000-sample dyadic bootstrap 95% CI of standardized β, and two-tailed bootstrap *p*-values are shown. “—” means the corresponding health literacy variable was excluded from the model. R² for patient and spouse equations are listed as patient outcome equation first, spouse outcome equation second. For sensitivity models, interdependence paths and predefined covariates of the final model were retained, without data-driven trimming of health literacy paths.

**Figure 2:**
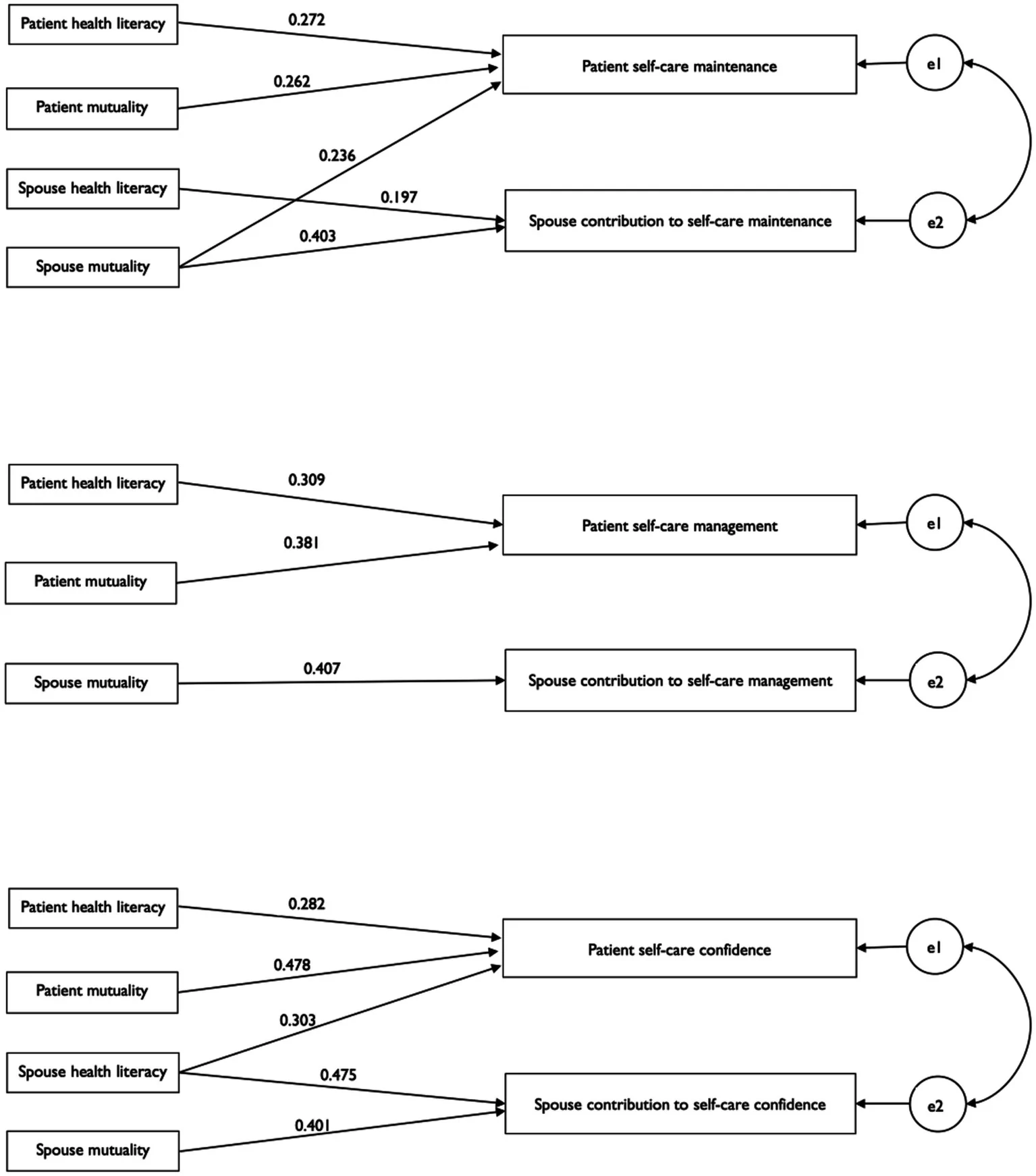
Actor-partner interdependence model examining the associations between patient and spouse health literacy, mutuality, and heart failure self-care outcomes across 3 dimensions: Self-care maintenance, self-care management, and self-care confidence. Arrows indicate statistically significant effects. Standardized coefficients (*β*) are shown.

The final APIM results indicated the following. In the maintenance model: patient health literacy and patient mutuality exerted positive actor effects on patient self-care maintenance; spouse mutuality exhibited a positive partner effect on patient self-care maintenance. Spouse health literacy and spouse mutuality had positive actor effects on spousal contribution to self-care maintenance. In the management model: patient health literacy and patient mutuality exerted positive actor effects on patient self-care management, and spouse mutuality had a positive actor effect on spouse contribution to self-care management. No significant partner effects remained after adjusting for prespecified covariates. However, the final model for the self-care management dimension did not meet conventional fit thresholds (*RMSEA* = 0.094, *CFI* = 0.873, *TLI* = 0.831). Several factors may account for this suboptimal fit. First, the self-care management scale is symptom-dependent, with generally low execution frequency among patients, resulting in a positively skewed score distribution that deviates from multivariate normality. Second, the core predictors (health literacy, mutuality, social support) explained less variance in self-care management than in the maintenance and confidence dimensions, as indicated by the lower *R^2^* values in regression analyses, suggesting that management behaviors may be more strongly driven by clinical factors such as symptom severity and access to care. Third, the sample size of 145 dyads provides limited statistical power for subscale-level structural equation modeling. Accordingly, path results for the management dimension should be interpreted cautiously as exploratory findings. In the confidence model: patient health literacy and patient mutuality exerted positive actor effects on patient self-care confidence; spouse health literacy showed a positive partner effect on patient self-care confidence. Spouse health literacy and spouse mutuality exerted positive actor effects on spouse contribution to self-care confidence. See Figure 2 for details. Paths involving social support were not retained in any of the three final models.

### 3.2 Qualitative results

**Figure 3:**
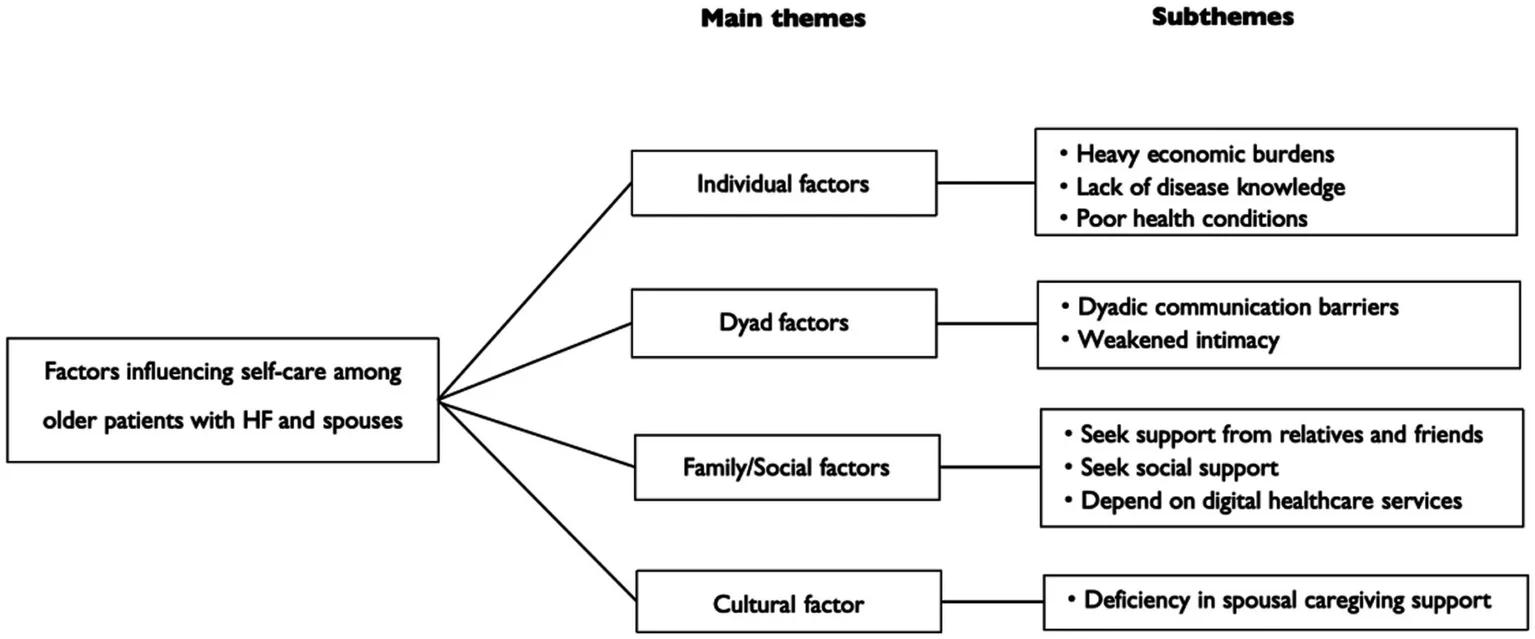
Factors influencing self-care among HF patients and their spouses, HF, heart failure.

A total of 10 dyads (20 participants) were enrolled in the study. Figure 3 illustrated four main themes and nine corresponding subthemes.

#### 3.2.1 Theme 1: Individual factors

##### 3.2.1.1 Heavy economic burden

HF is a chronic progressive disease characterized by a high rehospitalization rate. The costs incurred during hospitalization, whether for pharmacological treatments or device implantation, are substantial, imposing a significant economic burden on most families. Consequently, this financial burden impacts individual’s decision-making regarding health-related behaviors during the medical treatment process, including choices of surgical procedures, medication adherence, and frequency of follow-up appointments.

P01: “An artificial heart valve is expensive for our family. Due to receiving medical treatment in a different locality, we are required to pay the costs out of pocket upfront and subsequently return to our home area to complete the medical insurance reimbursement process.”

P09: “I take medication every day. I’ve been thinking about skipping pills whenever possible to save on costs.”

S04: “He has already been hospitalized twice this year. We postponed this hospitalization until we had borrowed the money from relatives.”

##### 3.2.1.2 Lack of disease knowledge

When patients and their spouses lack the essential knowledge and skills for self-care, they may be unable to effectively identify symptom changes and anticipate the consequences of disease progression. This inability often results in delays in implementing appropriate countermeasures, ultimately leading to delayed treatment and suboptimal outcomes.

P08: “The diuretic pills make my mouth dry, so I drink a lot of water. My swollen feet are getting worse.”

P09: “I feel chest tightness when I lie down, but sitting up provides some relief. I try to endure it whenever possible.”

S10: “The nurse said heart failure patients should follow a low-salt diet and monitor their daily weight, but I have not noticed him doing it at home. I do not think it matters much.”

##### 3.2.1.3 Poor health conditions

The HF condition often recurs, necessitating frequent visits to the clinics and hospitalizations, which consume a great deal of energy from both older patients and their spouses. As the cardiac function of older patients deteriorates, their physical strength and energy gradually decline. When spouses are in poor health because of their own medical conditions, the care they offer to the patients is often impaired to varying degrees. Interviews revealed that for older couples aged 60 and above, the health status of both partners had an impact on disease management, especially for those who had underlying chronic conditions.

P08: “I used to walk outside for half an hour every day, but I’m 70s now, it’s even difficult for me to go downstairs.”

S02: “I have hypertension. Whenever the weather turns cold, I often feel dizzy.”

S03: “I need to remind my wife to take her medication timely. When I was hospitalized due to illness, she often forgot the pills.”

#### 3.2.2 Theme 2: Dyad factors

##### 3.2.2.1 Dyadic communication barriers

Marital dyadic communication barriers have an adverse impact on dyad relationships and intensify disease-related stress. In contrast, effective communication promoted alignment in attitudes toward disease management and coping strategies (29). During the interview process, communication problems were identified in both patients and spouses.

P05: “He simply told me not to worry. He never discussed my condition with me. He made all the decisions regarding my treatment, which made me angry.”

S04: “We talked to each other happily, but now, he sticks to his views, I stick to mine. It always ends in arguments, and we can never reach any agreement. It’s so frustrating.”

##### 3.2.2.2 Weakened intimacy

Patients with HF frequently experience anxiety because of the recurrent nature of their condition, whereas their spouses suffer from physical and mental exhaustion due to the pressures of caregiving. This situation leads to a reduction in intimate interaction and a weakening of the relationship. The interviewed patients and their spouses reported that the reduced intimacy had a negative impact on self-care behaviors.

P04: “I’ve been in a bad mood ever since I got sick. I just want to be left alone. Sometimes I forget to take my pills.”

S06: “When I try to chat with him, he ignores me. I know that he’s feeling down due to his illness, but I’m also exhausted. Our relationship is no longer what it used to be.”

#### 3.2.3 Theme 3: Family/social factors

##### 3.2.3.1 Seek support from relatives and friends

For older patients with HF, apart from spousal care, the concern and support from other relatives and friends also play a positive role in the disease management process.

P01: “My son is always doing so many heartwarming little things. He has given me immense courage to face my illness.”

P08: “After I contracted this illness, I did not even want to leave the house. Fortunately, two old chess-playing friends often visit my home to accompany me. I follow the doctor’s advice, take my medicine on time, and watch my diet.”

##### 3.2.3.2 Seek social support

Older patients with HF seek multidimensional social support, including human resources, medical resources, and financial assistance. During the interviews, several respondents stated that strong social support led to more efficient utilization of healthcare services and eased the burden on family caregivers.

S03: “Last year, my wife suddenly experienced chest tightness while alone at home. She called the community center, and the staff quickly came to take her to the community hospital.”

S09: “My wife is frequently hospitalized due to heart failure. When her condition improves with medication at a general hospital, she returns to the community hospital for recovery. However, there were times when her condition suddenly deteriorated, she had to wait for a bed of general hospital. It would be much better if she could be directly transferred from the community hospital to a general hospital.”

##### 3.2.3.3 Depend on digital healthcare services

With the advancement of modern information technology, convenient and efficient digital healthcare has become increasingly popular among the general public.

P02: “Two days after being discharged with a pacemaker, I experienced swelling and pain in my wound. My daughter, who was busy with work, made an appointment with a wound care nurse online through her phone, it was quite convenient.”

P06: “There are always a large number of people in Chinese hospitals. Previously, I had to go to the hospital to see a doctor, it was time-consuming. Nowadays, it is also convenient for us older people to make an appointment via a mobile phone.”

S10: “My husband’s potassium levels are unstable, he often needs to have blood tests. Now, after having his blood drawn, we no longer need to wait at the hospital. Instead, we can check the test results in real-time on phones at home and consult a doctor online to adjust his medication.”

#### 3.2.4 Theme 4: Cultural factor

##### 3.2.4.1 Deficiency in spousal caregiving support

The interviews indicated that spousal caregivers not only shouldered caregiving responsibilities but also internalized emotional distress, for which adequate coping approaches were still unavailable.

S04: “Last Sunday, my son and daughter visited their father after his discharge. Everyone gathered around him. I was busy cooking all the time, the kitchen was stuffy. Throughout this period of caring for my husband, I’ve been exhausted, yet no one has shown any concern for me. It’s truly disheartening.”

S09: “My husband’s condition sometime makes me overwhelmed. I do not know how to deal with negative emotions. When I’m in a low mood, I simply lack the energy to take care of him.”

### 3.3 Integration of quantitative and qualitative results

Quantitative and qualitative findings were integrated within the framework of the TDIM. First, qualitative findings revealed that participants accessed healthcare-related support through digital channels, a form of support not captured by the SSRS. Combined with the null quantitative finding for SSRS-assessed social support, this observation provides a tentative hypothesis that social support measurement tools may need refinement for this population. Second, for the identified significant predictors (health literacy and mutuality), qualitative findings elaborated potential underlying pathways, such as dyadic communication barriers and insufficient disease knowledge. Third, qualitative analysis complemented the quantitative survey by uncovering additional unmeasured contextual factors, including economic burden, cross-regional medical insurance barriers, and cultural neglect of spousal caregivers. Table 20 presents a joint display mapping each TDIM dimension to corresponding quantitative results, matched qualitative themes, and types of integration.

**Table 20:** Integration of quantitative and qualitative findings grounded in the TDIM.

TDIM conceptual dimension	Key quantitative finding	Corresponding qualitative subtheme	Integrated interpretation
Individual factors	Age was negatively associated with self-care maintenance in both patients and spouses. Health literacy was positively associated with self-care maintenance and self-care confidence for both patients and spouses; health literacy was positively associated with patient self-care management. NYHA class was positively associated with patient self-care management, spouse contribution to self-care maintenance, and spouse contribution to self-care management.	Heavy out-of-pocket economic burden and medical reimbursement constraints Insufficient heart failure-specific knowledge and low disease awareness Comorbid chronic conditions and age-related functional decline	Confirmation: Quantitative actor effects of health literacy and age are consistent with qualitative accounts of knowledge gaps and functional limitations. Explanation: Regarding the positive correlation between NYHA class and self-care, possible hypotheses are selection bias, frequent clinical contact and increased vigilance. Complement: No statistically significant difference in financial pressure was observed in the quantitative study. Qualitative data reveal it as a barrier of self-care behaviors.
Dyad factors	Patient mutuality exerted positive actor effects on all three dimensions of patient self-care. Spouse mutuality exerted positive actor effects on all three dimensions of spouse contribution to self-care. Spouse mutuality exerted a positive partner effect on patient self-care maintenance.	Ineffective communication about symptom monitoring and treatment decisions Diminished marital intimacy and emotional support under long-term caregiving pressure	Confirmation: Qualitative findings align with the core TDIM assumption of dyadic interdependence, verifying that mutuality is associated with self-care. Explanation: Qualitative data unpack that poor dyadic communication and reduced intimacy erode mutual support, which in turn undermines shared illness management capacity.
Family/Social factors	Social support (SSRS total score) did not show a statistically significant difference in either the multiple linear regression model or the APIM analysis.	Rely on support from extended family and friends Rely on community-based heart failure care and follow-up services Rely on digital healthcare platforms as a compensatory support channel	Complement: In qualitative phase, participants’ reported use of digital healthcare services offers contextual insight into the null quantitative finding.
Cultural factor	No dedicated quantitative measurement of cultural.	Systemic invisibility of spousal caregivers in patient-centered clinical care settings	Complement: Qualitative result supplements that the traditional care culture is predominantly patient-centered, lacking sufficient support and attention for spousal caregivers. It complements culturally specific factor that is not captured by quantitative scales.

TDIM, Theory of Dyadic Illness Management; NYHA, New York Heart Association; APIM, Actor-Partner Interdependence Model; SSRS, Social Support Rating Scale.

## 4 Discussion

### 4.1 Factors associated with self-care based on theory of dyadic illness management

In this study, a comparative analysis of quantitative and qualitative findings has revealed both consistencies that mutually validate the results and new research insights. At the same time, this study extends the HF dyadic research in three ways. First, methodologically, prior studies in this field have relied on cross-sectional quantitative designs (18, 19) or qualitative study (30). In this study, the explanatory sequential mixed-methods approach complement statistical associations with in-depth qualitative evidence, enabling us to explore complex factors in chronic disease management. Second, conceptually, our study focuses exclusively on spousal dyads. Spousal caregivers represent a distinct subgroup with longer shared care histories and higher interdependence. Third, this study generates hypotheses for potential context-specific additions to the TDIM, and provides preliminary contextual evidence. Notably, the supplementary dimensions identified in this study (including digital healthcare access, regional care culture, and medical insurance-related economic burden) are exploratory observations from our sample.

#### 4.1.1 Individual factors

In this study, quantitative results indicate an association between age and self-care maintenance of patients and their spouses, while qualitative findings suggest that the majority of older adults suffer from chronic diseases, with poorer health status correlating to worse self-care performance. Advancing age leads to a decline in physiological function and an increased likelihood of illness, resulting in physical frailty, which in turn affected self-care (31). Therefore, compared with younger and middle-aged patients, we need to pay more attention to the older people and their self-care behaviors.

The quantitative results showed that higher NYHA classification significantly related to better self-care management, which differed from findings in the previous study (32). Combined with the interviews conducted in the subsequent qualitative study, several potential explanations may account for this association. First, there may be a selection bias: our sample was recruited from follow-up clinics or prior to discharge, and patients with severe HF who have poor self-care are more likely to present via emergency admission or be lost to follow-up, thus being underrepresented in this study. Second, patients with more severe symptoms and higher NYHA classification typically have more frequent clinical contacts and receive more intensive heart failure education during hospitalizations and outpatient visits, which may increase their disease awareness and self-care. Third, it is also possible that patients who have experienced worsening symptoms have become more proactive in self-care management in response to their condition. Longitudinal cohort designs are needed in future research to clarify the dynamic relationship between disease severity and self-care over time.

The APIM analysis identified a role-specific cross-partner statistical association: higher spouse-reported heart failure health literacy was associated with greater patient self-care confidence. This finding suggests that when spouses have a higher level of health literacy, it may encourage patients to engage in self-care with greater confidence. In this study, as patient and spouse health literacy were measured using two different population-specific instruments, the health literacy’s actor and partner effects cannot be directly compared. The observed partner effect of health literacy may be confounded by compositional differences between the two instruments, so it cannot be interpreted as a pure dyadic spillover effect. Future research is suggested to use matched measurement tools to further verify this correlation. The qualitative findings revealed that a lack of cognition about HF prevented patients from effectively monitoring changes in their symptoms, leading to delays in seeking medical care and, consequently, a worsening of their condition. Health literacy encompasses not only knowledge and skills regarding diseases but also the capacity to access health services and make health-related decisions. It served as a crucial enabling factor in disease self-care (33). Individuals with higher levels of health literacy exhibit improved learning ability, communication skills, and a proactive attitude, thus acquiring more knowledge and competencies (34). When individuals recognize the importance of self-care, they can achieve a higher quality of life through self-care behaviors. Therefore, it is necessary to enhance health literacy of both older patients and their spouses.

Furthermore, quantitative analysis found no statistically significant association between self-reported financial pressure and self-care. Complementing this quantitative finding, qualitative interviews identified perceived economic barriers that shape self-care decision-making in practice, thereby providing possible explanations. As a chronic and progressive condition, HF imposes a continuous financial drain on families. Financial strain is not merely a background variable, it directly dictates clinical behaviors. In the interviews, participants P09 and S04 reported that they weakened self-care behaviors, such as reducing medication frequency or delaying follow-up visits to mitigate the family’s economic pressure (35). Older adults in China tend to prioritize household savings over personal healthcare spending. If they face financial difficulties, they are willing to cut back on their personal spending. Meanwhile, as the primary caregiver, the spouse is facing the dual burden of household living expenses and medical costs. This pressure can easily trigger communication conflicts and emotional burnout within the dyadic relationship (36). Although the current medical insurance payment reform has expanded coverage, there are still gaps in implementation regarding the convenience of cross-regional settlement. These findings, drawn from participants’ lived experiences of economic strain, offer contextual implications for policy optimization.

#### 4.1.2 Dyad factors

The quantitative study found that higher-level mutuality was associated with better self-care ability. The qualitative findings revealed that communication barriers and diminished intimacy within the dyadic relationship negatively affected disease self-care. Mutuality is defined as the positive quality of the relationship between the couple (26). Mutuality consists of four dimensions: love and affection, shared pleasurable activities, shared values, and reciprocity (16, 17). Patients with higher-level mutuality experienced fewer negative emotions during treatment, showed better treatment adherence (37). Good communication and a strong relationship between older couples make the spouse more willing to provide care and support to the patients. Notably, mutuality was positively associated with self-care outcomes in both patients and their spouses; in contrast, general social support did not show statistical significance. This highlights that mutuality between the couple plays a significant role in heart failure self-care. Clinically, these findings advocate for implementing dyadic interventions focused on enhancing relational mutuality.

#### 4.1.3 Family/social factors

In the quantitative phase, social support is assessed using the SSRS, which focuses on care and assistance from family members, neighbors, friends, and employers. However, there was no statistically significant association. Further qualitative phase exploration revealed that participants’ perceptions of social support in disease management extended beyond the scale items, which presented as one plausible contextual insight that may help interpret the null finding. However, this hypothesis requires verification in future studies. The qualitative results uncovered a reliance on digital healthcare services for older people. Participant P02 utilized online platforms to secure wound care nursing services and participant S10 monitored serum potassium levels for real-time online medication adjustments, illustrating how digital medical service compensated for the inadequacy of traditional social support dimensions. The interviews revealed that older adults were willing to use digital medical services, provided they are well-designed and user-friendly, because such digital solutions indeed offered convenience and efficiency. The convenient digital healthcare infrastructure and proficient digital health literacy enhances symptom perception levels for both members of the dyad, which is a critical cognitive prerequisite for triggering subsequent self-care behaviors. These observations were consistent with the research by Yoong et al. (12), which demonstrated that mobile health interventions effectively reduced hospital readmission rates and improved health-related quality of life in heart failure populations. However, it is important to note that not all older adults can benefit equally from digital healthcare services. Older adults with limited digital skills, visual/hearing impairment, or cognitive decline face operational barriers to using mobile health applications, which may prevent them from benefiting from digital services and could exacerbate health disparities (38). The adoption of digital services by older adults is influenced by their perceptions of ease of use and usefulness (39). Digital healthcare penetration varies across China. We note that since our sample was recruited from a tertiary hospital in Zhejiang which is a highly developed region with digital health infrastructure, our findings may overestimate the accessibility and usability of digital healthcare for populations in economically underdeveloped regions. Rural and socioeconomically underdeveloped regions face inadequate network infrastructure and limited access to smart devices. Similarly, compared with tertiary hospitals, primary care facilities have a weaker capacity to provide digital healthcare services. Therefore, older adults in these settings cannot access digital health services as readily as those in developed coastal areas. Furthermore, the acceptance of digital healthcare services is influenced by regional cultural norms and patterns of intergenerational support. Older adults in regions with stronger traditional family care norms may be less inclined to use digital healthcare services independently, preferring instead to rely on their children for assistance. Participants’ accounts of digital healthcare use offer contextual insights for service optimization. Future efforts may prioritize expanding digital healthcare infrastructure in underdeveloped regions and primary care settings, and developing age-friendly digital tools for vulnerable groups, while retaining offline care alternatives.

#### 4.1.4 Cultural factor

Regarding the care culture, spouses S04 and S09 stated that they felt overlooked and lacked support while caring for the patients. In the clinical setting where this study was conducted, we observed a tendency for healthcare professionals and family members to focus primarily on the patient as the sole recipient of care, with relatively less attention given to the spouse as a caregiver. This observation is consistent with the patient-centered care paradigm commonly practiced in clinical settings, which is not conducive to dyadic relationships and disease management (22). We acknowledge that this finding is based on a limited sample of spouses and should not be generalized as a universal characteristic of Chinese culture. Nevertheless, the finding highlights the necessity of addressing the caregiving needs of both members in clinical practice. We suggest that future research investigate whether this phenomenon is prevalent across different geographical and cultural contexts.

### 4.2 Limitations

This study has several limitations. The findings on digital healthcare and spousal caregiver neglect were obtained from a small sample of participants at a single tertiary hospital. Future multi-center studies conducted across diverse geographic and institutional settings are required to verify the generalizability of these observations. We chose versions of the SCHFI 6.2 and CC-SCHFI 1 to measure self-care, as the Chinese validated versions of these two scales were the most widely utilized in clinical practice at the time. These scales do not include a mental health dimension, this omission may have led to an incomplete assessment. The traditional SSRS was developed prior to the widespread adoption of digital health services. Future studies should employ updated versions of HF self-care scales and completed social support scales. Data in the quantitative phase were collected through self-report questionnaires, which may be subject to recall bias and social desirability bias. Health literacy were measured using two different population-specific instruments. Therefore, the observed partner effect of health literacy cannot be interpreted as a pure dyadic spillover effect. Within the APIM framework, non-significant partner effects cannot be interpreted as a lack of dyadic interaction. Limited statistical power may prevent the detection of small or moderate partner effects in the current sample. Owing to unsatisfactory model fit, the APIM findings for the management model cannot serve as conclusive evidence and are merely exploratory for future study. Finally, as a cross-sectional study, this design precludes establishing causal relationships between variables and outcomes.

## 5 Conclusion

This explanatory sequential mixed-methods study systematically identified the factors influencing self-care in older patients with HF and their spouses using APIM dyadic analysis. Health literacy and mutuality play significant roles in self-care for heart failure among older adults. Different dimensions of self-care are influenced by distinct factors and exhibit unique dyadic interaction patterns. Clinical interventions should develop patient-spouse dyadic support strategies tailored specifically to self-care maintenance, self-care management, and self-care confidence. Furthermore, effective dyadic interventions should fully incorporate age-friendly digital health services, health insurance support, and sociocultural caregiving patterns. Future multi-center longitudinal and intervention studies are warranted to validate these hypothesized mechanistic pathways and refine collaborative self-care strategies for heart failure patient–spouse dyads.

## Data Availability

The datasets presented in this article are not readily available because the quantitative dataset analyzed during this study is not publicly available due to ethical restrictions (participant confidentiality and institutional data protection policies). The qualitative interview guide is provided in Table 1. Deidentified qualitative transcripts are not publicly available to protect participant privacy. Qualified researchers upon reasonable request and with appropriate confidentiality agreements, are available from the corresponding author. Requests to access the datasets should be directed to YZ, zhuangyy@zju.edu.com.
